# Assessing Discriminative Performance at External Validation of Clinical Prediction Models

**DOI:** 10.1371/journal.pone.0148820

**Published:** 2016-02-16

**Authors:** Daan Nieboer, Tjeerd van der Ploeg, Ewout W. Steyerberg

**Affiliations:** 1 Department of Public Health, Erasmus MC—University medical center, Rotterdam, the Netherlands; 2 Department of Science, Medical Center Alkmaar/Inholland University, Alkmaar, the Netherlands; University of Oxford, UNITED KINGDOM

## Abstract

**Introduction:**

External validation studies are essential to study the generalizability of prediction models. Recently a permutation test, focusing on discrimination as quantified by the *c*-statistic, was proposed to judge whether a prediction model is transportable to a new setting. We aimed to evaluate this test and compare it to previously proposed procedures to judge any changes in *c*-statistic from development to external validation setting.

**Methods:**

We compared the use of the permutation test to the use of benchmark values of the *c*-statistic following from a previously proposed framework to judge transportability of a prediction model. In a simulation study we developed a prediction model with logistic regression on a development set and validated them in the validation set. We concentrated on two scenarios: 1) the case-mix was more heterogeneous and predictor effects were weaker in the validation set compared to the development set, and 2) the case-mix was less heterogeneous in the validation set and predictor effects were identical in the validation and development set. Furthermore we illustrated the methods in a case study using 15 datasets of patients suffering from traumatic brain injury.

**Results:**

The permutation test indicated that the validation and development set were homogenous in scenario 1 (in almost all simulated samples) and heterogeneous in scenario 2 (in 17%-39% of simulated samples). Previously proposed benchmark values of the *c*-statistic and the standard deviation of the linear predictors correctly pointed at the more heterogeneous case-mix in scenario 1 and the less heterogeneous case-mix in scenario 2.

**Conclusion:**

The recently proposed permutation test may provide misleading results when externally validating prediction models in the presence of case-mix differences between the development and validation population. To correctly interpret the *c*-statistic found at external validation it is crucial to disentangle case-mix differences from incorrect regression coefficients.

## Introduction

Clinical prediction models receive increasing attention for medical practice and research. After the development of a prediction model, an external validation study is essential to explore whether predictions done by the model are valid in a new population [1,2]. The discriminative ability of prediction models is often quantified using a concordance (*c*) statistic [3]. The *c*-statistic measures whether a prediction model can discriminate between patients with and without the outcome of interest. For logistic regression models the *c*-statistic is equivalent to the area under the receiver operating characteristic (ROC) curve (AUC) [4].

External validation studies are considered the stronger tests for a model compared to internal validation procedures such as cross-validation or bootstrap resampling [5]. The possible differences between the validation and development setting make an external validation study a test of ‘transportability’ of a prediction model [6]. If the validation population contains similar patients as the development population, the external validation study could merely be considered a test of the ‘reproducibility’ of a prediction model. Reproducibility refers to the ability of a prediction model to give valid predictions in a population very similar to the development population, whilst transportability refers to the ability to give valid predictions in populations that are related to but different from the development population [6]. Typical examples of tests of transportability are assessment of model performance across different geographical regions or in different time periods.

We previously proposed a framework to identify if an external validation study investigated the reproducibility or transportability of a prediction model [7]. This framework consists of three steps, 1) investigate the relatedness of the development and validation population, 2) validation of the prediction model in the new population, and 3) interpreting the results found at step 2 using the results from step 1. To assess the relatedness between the development and validation sample we proposed a membership model, i.e. a model predicting whether a patient is from the development or validation sample. Moreover, we suggested to compare the standard deviation of the linear predictors between development and validation samples, where the linear predictor is the linear combination of the regression coefficients from the model and the covariate values in the development and validation samples respectively.

Recently a permutation test was introduced with the aim to consolidate step 1 & 2 into a single step [8]. The permutation test tests the hypothesis that the development and validation population are homogeneous. The permutation test obtains a p-value by judging the change in *c*-statistics of the model between the development and validation samples. The permutation test assesses the degree of homology between the development and validation sets. When the permutation test gives a p-value below a pre-specified threshold, typically 0.05, the hypothesis that the development and validation samples are homogeneous is rejected. The claim was that the model may then not be directly transported to the validation population without further revision or updating [8].

Previous research has shown that the *c*-statistic does not only depend upon the validity of the prediction model, i.e. correctness of the regression coefficients, but also on the case-mix, i.e. the heterogeneity between patients in the population [9]. A measure of the case-mix heterogeneity is the standard deviation of the linear predictor in a sample. Benchmark values of the *c*-statistic have also been developed to disentangle case-mix effects from the effects of incorrect regression coefficients [10]. One such benchmark value is called the model based *c*-statistic (mbc). This is the expected *c*-statistic in a population given that the predictions made by a model are perfectly valid.

We aimed to evaluate the usefulness of the recently proposed permutation test in relation to the previously proposed framework. Specifically, we compare the conclusions from this test with conclusions drawn using earlier proposed measures, i.e. the standard deviation of the linear predictor and benchmark values of the *c*-statistic. We evaluated the different measures using a simulation study and in a case study using 15 datasets containing patients suffering from traumatic brain injury (TBI).

## Methods

We considered three strategies to judge a change in *c*-statistic when externally validating a prediction model: a permutation test; the standard deviation of the linear predictor; and benchmark values of the *c*-statistic.

### Simulation study

To assess the performance of the proposed permutation test we conducted a simulation study. In the simulation we varied the case-mix differences and predictor effects between the development and validation population, we also varied the sample sizes available for development and validation. In our simulation study we generated a development set **D** and validation set **V** using the following model:

$$y_{ij}∼\text{bernoulli}\left(\pi_{ij}\right),$$

$$\pi_{ij}={\text{logit}}^{-1}\left(\beta_{j}x_{ij}\right),$$

$$x_{ij}\sim N\left(0,\sigma_{j}^{2}\right),$$

Where *i* denotes the patient number and *j* ∈ {*D*, *V*} indicates whether the patient belongs to the development or validation set.

We distinguished three scenarios. In the first scenario we assumed that the case-mix distribution and predictor effects in the development and validation dataset are homogeneous. In the second situation we assumed that the case-mix is more heterogeneous and predictor effects were weaker in the validation set compared to the development set. In the third situation we assumed that the case-mix was less heterogeneous in the validation set as compared to the development set, but that the predictor effects were similar. The different values of the parameters in the different situations are shown in Table 1.

**Table 1:** Parameters of the different scenarios in the simulation study.

	SD predictor development population (σ_D_)	SD predictor validation population (σ_V_)	Coefficient development population (β_D_)	Coefficient validation population (β_V_)
Homogeneous populations	1	1	3	3
Different case-mix & predictor effects	1	1.5	3	2
Different case-mix & same predictor effects	1	0.75	3	3

SD: standard deviation

For each situation we generated development and validation sets containing 40, 100 or 200 patients, resulting in 9 scenarios in total. We developed a prediction model in the development set using logistic regression and validated the resulting model in the validation set. Subsequently we calculated the standard deviation of the linear predictor in the development and validation sets, the ratio of the standard deviation of the linear predictor in the validation and development set, two benchmark values for the *c*-statistic and performed the permutation test. R-scripts used for the simulation study are available as supplementary material (S1 Appendix).

### Case study

Our case study uses data from the IMPACT database [11]. This database contains data from 15 studies with patients suffering from traumatic brain injury (TBI). This database was previously used to develop a prediction model predicting 6-month mortality and unfavorable outcome using patient characteristics such as age, motor score and pupillary reactivity [12]. In our case study we developed a model predicting 6 month mortality using the on the international arm of the Tirilizad trial [13]. The prediction model contained the predictors age, motor score and pupillary reactivity. We subsequently validated the developed model in the remaining 14 studies and judged the change in *c*-statistic observed at development and at external validation using the ratio of the standard deviation of the linear predictor in the validation and development set, benchmark values of the *c*-statistic, and the permutation test. All statistical analyses were done in R 3.1.2 [14].

### Permutation test

The permutation test was developed to test the null hypothesis that the development and validation populations are homogeneous. When the null-hypothesis is not rejected it should be safe to transport the prediction model from the development to the validation population. We then claim that the model is valid in the validation population The permutation test starts with calculating the observed *c*-statistic at external validation, denoted by *c*^V^, of a prediction model. The *c*-statistic is calculated by comparing predictions from the model to observed outcomes. Subsequently patients are randomly permuted between the development and validation population. A prediction model is developed on the permuted development set and the *c*-statistic of this model is estimated in the permuted validation population. This process is repeated *k* times. The p-value of the permutation test is given by the proportion of times that the *c*-statistic of the model developed on the permuted development set was smaller than *c*^V^. Whenever the p-value is below a prespecified threshold, typically 0.05, the null hypothesis is rejected that the development and validation population are homogeneous. The prediction model should then be updated before being transported to the validation population. For our simulation and case study we used a value of *k* equal to 1,000.

### Measures of case-mix

A direct way to investigate the difference in case-mix between the development and validation population is to compare the standard deviation (SD) of the linear predictor of the prediction model in the development and validation population. The linear predictor is the linear combination of the regression coefficients from the model and the covariate values in the development and validation samples respectively:

$${lp}_{D}=X_{D}\beta_{D};\;{lp}_{V}=X_{V}\beta_{D}.$$

A population with a more heterogeneous case-mix has a higher SD of the linear predictor compared to a more homogeneous case-mix.

### Benchmark values

The discriminative ability of a prediction model at external validation can be influenced by both the correctness of regression coefficients and the case mix heterogeneity in the validation sample. This was a key point in our proposed framework and other work [7,10]. Since differences in case-mix have no impact on the validity of the prediction model it is important to distinguish between the influence of incorrect regression coefficients and case-mix. Therefore, two benchmark values of the *c*-statistic were proposed, the model based *c*-statistic (mbc) and the *c*-statistic obtained by refitting the model in the validation sample (*c*^refitted^) [10]. The mbc is the expected *c*-statistic in a population given that the prediction model is correct. Differences between the mbc and observed c-statistic at external validation (*c*^V^) indicate the extent of poor model fit independent of differences in case-mix between development and validation samples. The mbc can be obtained by first calculating the predicted probability for each patient in the validation sample and subsequently generating a new outcome value based on this probability [10]. To ensure stable estimates of the benchmark values at least 100 repetitions for each subject are required. The refitted *c*-statistic (*c*^refitted^) gives an upper bound on the performance of the model in the validation population, if the regression coefficients from the prediction model are perfectly valid. Comparison of *c*^V^ and *c*^refitted^ reflects the influence of incorrect regression coefficients, given a similar case-mix as in the validation population. The mbc uses the regression coefficients from the prediction model developed in the validation sample, while *c*^refitted^ uses regression coefficients from the validation sample. Interpretation of *c*^V^ is possible by considering the combination of the validity of the regression coefficients (as learned from comparison to *c*^refitted^) and the case-mix (difference to development sample learned from comparison to mbc).

## Results

### Simulation study

In the scenario where the development and validation population were homogeneous, and the development and validation sets were relatively small, the median *c*^D^ and *c*^V^ were both 0.92 (Table 2). Benchmark values of the *c*-statistic indicated that the regression coefficients of the model and case-mix between the development and validation sets were similar. Since the *c*-statistic is a rank based measure, by definition *c*^V^ and *c*^refitted^ were equal to each other in our simulations. The standard deviation of the linear predictor was similar in the development and validation sets, correctly indicating that the case-mix was similar in both samples, the median ratio of both standard deviations was close to 1. The permutation test rejected the null hypothesis of homogeneity between the validation and development sets in approximately 5% of the generated samples in the simulation study. Changing the sample size of the development and validation sets yielded similar results, however the interquartile range became somewhat smaller.

**Table 2:** Results from the simulation study, median and inter-quartile range of 1000 simulations is shown for *c*-statistics and standard deviations. Proportion of samples where the permutation test rejected the null hypothesis is shown.

	*c*^D^	*c*^V^	SD lp_D_	SD lp_V_	Ratio SD lp_V_ and SD lp_D_	mbc	*c*^refitted^	Proportion samples null-hypothesis rejected
Same case-mix and predictor effects								
Small sample (epv: 20)	0.92 (0.89–0.95)	0.92 (0.90–0.95)	3.14 (2.52–4.30)	3.16 (2.57–4.14)	1.01 (0.91–1.12)	0.93 (0.90–0.95)	0.92 (0.90–0.95)	0.06
Medium sample size (epv: 50)	0.92 (0.90–0.94)	0.92 (0.90–0.94)	3.08 (2.63–3.52)	3.05 (2.65–3.55)	1.00 (0.93–1.07)	0.92 (0.90–0.94)	0.92 (0.90–0.94)	0.05
Large sample size (epv: 100)	0.92 (0.91–0.93)	0.92 (0.91–0.93)	3.04 (2.75–3.37)	3.04 (2.74–3.39)	0.99 (0.95–1.05)	0.92 (0.91–0.93)	0.92 (0.91–0.93)	0.06
Different case-mix and predictor effects								
Small sample (epv: 20)	0.92 (0.89–0.95)	0.92 (0.89–0.95)	3.23 (2.58–4.14)	4.85 (3.89–6.22)	1.49 (1.35–1.67)	0.96 (0.95–0.98)	0.92 (0.90–0.95)	0.03
Medium sample size (epv: 50)	0.92 (0.90–0.94)	0.92 (0.90–0.94)	2.06 (2.67–3.67)	4.56 (4.00–5.32)	1.50 (1.41–1.60)	0.96 (0.95–0.97)	0.92 (0.90–0.94)	0.01
Large sample size (epv: 100)	0.92 (0.91–0.93)	0.92 (0.91–0.94)	3.04 (2.75–3.37)	4.56 (4.12–5.06)	1.50 (1.43–1.57)	0.96 (0.95–0.97)	0.92 (0.91–0.94)	0.02
Different case-mix and same predictor effexts								
Small sample (epv: 20)	0.92 (0.89–0.95)	0.88 (0.85–0.92)	3.22 (2.55–4.23)	2.42 (1.90–3.18)	0.75 (0.67–0.84)	0.89 (0.86–0.93)	0.88 (0.85–0.92)	0.18
Medium sample size (epv: 50)	0.92 (0.90–0.94)	0.88 (0.86–0.90)	3.08 (2.63–3.61)	2.30 (2.01–2.70)	0.75 (0.70–0.80)	0.89 (0.86–0.91)	0.88 (0.86–0.90)	0.25
Large sample size (epv: 100)	0.92 (0.91–0.93)	0.88 (0.86–0.90)	3.03 (2.74–3.37)	2.27 (2.06–2.52)	0.75 (0.71–0.79)	0.88 (0.87–0.90)	0.88 (0.86–0.90)	0.39

*c*^D^: *c*-statistic of the model in the development population

*c*^V^: *c*-statistic at external validation

SD lp_D_: standard deviation linear predictor in development population

SD lp_V_: standard deviation linear predictor in validation population

mbc: model based *c*-statistic

*c*^refitted^: *c*-statistic of model refitted in the validation population

When both the case-mix and regression coefficients in the development and validation population were different the median *c*^D^ and *c*^V^ were both equal to 0.92. The median mbc was equal to 0.96, by definition *c*^refitted^ was equal to 0.92. This indicated that although *c*^V^ did not change, a substantially higher *c*-statistic was expected if the regression coefficients of the original model had been correct. With small sample sizes, the median standard deviation of the linear predictor was 3.23 in the development set and somewhat larger (4.85) in the validation set, indicating a more heterogeneous case-mix. The median ratio of the standard deviation of the linear predictor in the validation and development sample also indicated that the case mix was more heterogeneous in the validation population (1.50). The permutation test rejected the null hypothesis in 3% of the generated samples. Results using larger sample sizes were similar, however the interquartile range became somewhat smaller.

In the third scenario, the case-mix in the validation population was less heterogeneous compared to the development population. Using small sample sizes for model development and validation, the median *c*^D^ was 0.92 and the median *c*^V^ was 0.88. The median mbc was equal to 0.89 and median *c*^refitted^ was 0.88, indicating that the drop in *c*-statistic between the development and validation set was due to a less heterogeneous case-mix in the validation set rather than incorrect regression coefficients. The median standard deviation of the linear predictor in the development population was 3.22 and somewhat smaller (2.42) in the validation set, indicating that the case-mix distribution was less heterogeneous in the validation set, which was confirmed by the median ratio of the standard deviations (0.75). The permutation test rejected the null hypothesis of homogeneous population in approximately 18% of the cases. Increasing the available sample sizes of the development and validation sets showed similar results, except for the permutation test where the proportion of samples where the null hypothesis was rejected increased to 39% as the available sample size increased, reflecting more statistical power. Again the inter-quartile range became somewhat smaller.

### Case study

The prediction model developed in the international arm of the Tirilizad trial had a *c*-statistic of 0.71 [0.67–0.74 95%CI]. The standard deviation of the linear predictor at development was equal to 0.80. When the model was externally validated the *c*-statistic ranged between 0.64 and 0.85 (Table 3). If the standard deviation of the linear predictor was larger in the validation sample than in the development sample, then the model based *c*-statistic (mbc) was larger than the *c*-statistic at development. This reflected the wider spread of the risk distributions. The permutation test indicated evidence of heterogeneity between the development and validation sample in 4 out of 14 validations. However, the mbc and the standard deviation of the linear predictor indicate that the decrease in *c*-statistic in the SLIN study was mainly attributable to a less heterogeneous case-mix distribution, rather than incorrect regression coefficients. The permutation test indicated no evidence of heterogeneity in 10 out of the 14 validation studies. Evaluation of the mbc and standard deviation of the linear predictor led to the same conclusion for the TIUS and PEG studies. In the other 8 cases there was a substantial influence of incorrect regression coefficients on the observed *c*-statistic at external validation, indicating that the model was not transportable to these settings.

**Table 3:** External validation results of the model predicting 6-month mortality in TBI patients using age, motor score and pupillary reactivity.

Study	*c*^V^	mbc	SD lp_V_	Ratio SD lp_V_ and SD lp_D_	*c*^refittted^	p-value permutation test
TINT	0.71^1^	-	-	-	-	-
TIUS	0.74	0.73	0.87	1.09	0.74	1.00
SLIN	0.68	0.69	0.71	0.89	0.68	0.00
SAP	0.69	0.74	0.95	1.19	0.74	0.00
PEG	0.76	0.78	1.17	1.46	0.77	1.00
HIT I	0.72	0.77	1.12	1.40	0.79	0.90
UK4	0.81	0.78	1.16	1.45	0.83	1.00
TCDB	0.82	0.80	1.25	1.56	0.83	1.00
SKB	0.68	0.75	1.01	1.26	0.72	0.35
EBIC	0.83	0.79	1.24	1.55	0.85	1.00
HIT II	0.69	0.77	1.10	1.38	0.73	0.00
NABIS	0.69	0.76	1.04	1.30	0.72	0.55
CSTAT	0.75	0.72	0.86	1.08	0.77	1.00
PHARMOS	0.64	0.70	0.76	0.95	0.66	0.00
APOE	0.85	0.73	0.86	1.08	0.85	1.00

^1^*c*-statistic of the model at development

*c*^V^: *c*-statistic observed at external validation

mbc: model based *c*-statistic

SD lp_V_: standard deviation of the linear predictor in the validation data

SD lp_D_: standard deviation of the linear predictor in the development data

*c*^refitted^: *c*-statistic of the prediction model refitted in the validation data

## Discussion

This study illustrated how two separate phenomena determine differences in observed discriminative ability, i.e. the *c*-statistic, between development and validation settings. Case-mix and the correctness of regression coefficients both influence the *c*-statistic of a prediction model when applied in a new datasets. Attempts to provide a single summary test for differences in *c*-statistic are therefore misleading. The recently proposed permutation test incorrectly concluded that the development and validation population were not homogeneous in the scenario with different case-mix but similar predictor effects. Conversely the permutation test concluded that development and validation population were homogeneous when the case-mix was more heterogeneous but predictor effects were weaker. Similar patterns were observed in the case study.

The permutation test aimed to consolidate the first two steps in the framework proposed by Debray et al. [7], by judging the heterogeneity between development and validation population using the change in *c*-statistic of the prediction model. The *c*-statistic however does not only depend on whether a prediction model gives valid predictions, but also on the case-mix in the underlying population; that was the key point in the framework by Debray. The permutation test does not take these case-mix differences into account and may break down when these are present.

When validating the IMPACT prediction model, predicting 6-month mortality of patients suffering from traumatic brain injury, it was noted that the *c*-statistic at external validation was higher in datasets from observational studies compared to the *c*-statistic found when validating in datasets from randomized controlled trials [15]. These differences were attributed to the wider enrollment criteria in the observational studies compared to trials, leading to a more heterogeneous case-mix in the observational studies compared to the trials. Similarly, a recent review found higher *c*-statistic values in some validation studies than in the development studies, again suggesting that more heterogeneity at validation is well possible [16]. The overall pattern in this review was a lower performance at validation than expected, reflecting overoptimism and overfitting at model development [4].

At external validation the performance of a prediction model is assessed using data not used at model development. Here we focused on judging the change in *c*-statistic of the prediction model at external validation. Validation studies however should also aim to assess other model properties, in particular the calibration of a prediction model. Calibration refers to the agreement between predicted probabilities and observed outcomes. It can adequately be assessed using recalibration parameters, and graphically using calibration plots [4,17].

The standard deviation of the linear predictor is a simple measure of case-mix heterogeneity in a dataset. When the distribution of the linear predictor is skewed the standard deviation may not be appropriate as a measure of case-mix heterogeneity. We note however that the distribution of the linear predictor is often close to a normal distribution [18]. At external validation the distribution of the linear predictor should be assessed graphically in a ‘validation’ plot [19,20].In sum, the proposed permutation test does not take case-mix differences into account and can therefore give misleading results in the presence of case-mix differences. The permutation test therefore is only useful when there are no case-mix differences between the development and validation set. Case-mix differences between development and validation setting can readily be detected by simple summary measures such as the variance of the linear predictor or benchmark values of the *c*-statistic. To judge the change in c-statistic of a prediction model at external validation it is crucial to disentangle the effects of incorrect regression coefficients from differences in case-mix heterogeneity between the development and validation setting.

## Supporting Information

S1 Appendix R-script used for the simulation study. (R)
